# Polypoidal Choroidal Vasculopathy Complicated by Red Blood Cell-Coated Intraocular Lens: A Case Report

**DOI:** 10.7759/cureus.69639

**Published:** 2024-09-18

**Authors:** Aisha Sawazono, Ryoh Funatsu, Hiroto Terasaki, Naohisa Mihara, Taiji Sakamoto

**Affiliations:** 1 Ophthalmology, Kagoshima University Graduate School of Medical and Dental Sciences, Kagoshima, JPN

**Keywords:** age-related macular degeneration, intraocular lense, optical coherence tomography, polypoidal choroidal vasculopathy, rbc-coated iol

## Abstract

This study aimed to characterize the detailed multi-modal imaging findings of red blood cell (RBC)-coated intraocular lenses (IOLs). A 68-year-old patient with polypoidal choroidal vasculopathy underwent vitrectomy for subretinal and vitreous hemorrhage. Subsequently, RBC-coated IOL was diagnosed. The iris and IOL surface exhibited a reddish discoloration, while the fundus was completely obscured by slit-lamp examination and ultra-widefield scanning laser ophthalmoscopy. However, posterior segment optical coherence tomography (OCT) allowed visualization of retinal structures. Anterior segment OCT revealed no opacity in the optic part of the IOL in either eye, with comparable findings between both eyes. Given the high absorption spectrum of blood in the visible light range and its minimum absorption at approximately 1100 nm, RBC-coated IOLs may minimally affect anterior and posterior segment OCT images. Conversely, they significantly impair slit-lamp examination and direct fundus visualization. The discrepancy in imaging outcomes between fundus image and OCT could be a characteristic feature of RBC-coated IOLs. This may serve as a characteristic of RBC-coated IOLs. In cases of suspected IOL opacification or RBC-coated IOL following vitreous hemorrhage, anterior segment OCT can evaluate the IOL optic clarity. Additionally, comparing image quality between fundus photographs and posterior segment OCT may provide valuable diagnostic information.

## Introduction

Polypoidal choroidal vasculopathy (PCV) is a subtype of neovascular age-related macular degeneration characterized by distinctive polypod lesions within macular neovascularization [1]. This condition can lead to severe vision loss due to massive hemorrhage from polyp rupture, resulting in subretinal and breakthrough vitreous hemorrhage [1-3]. Kim et al. reported that PCV cases complicated by breakthrough vitreous hemorrhage are occasionally associated with red blood cell (RBC)-coated intraocular lenses (IOL) [4]. However, detailed clinical findings of this phenomenon remain limited.

Kim et al. noted that IOL opacity and RBC-coated IOLs are prone to misdiagnosis [4]. While IOL opacity typically requires lens replacement, RBC-coated IOLs often resolve spontaneously within six months to a year [4-7]. Accurate differentiation is crucial, especially in patients who have undergone pars plana vitrectomy with endotamponade, as this procedure is a risk factor for IOL opacification [8]. Therefore, determining the characteristic features of RBC-coated IOLs is essential for accurate diagnosis and appropriate management.

This case report presents a PCV patient who developed RBC-coated IOL following vitrectomy for breakthrough vitreous hemorrhage. We provide a comprehensive analysis of multi-modal imaging findings associated with this complication.

## Case presentation

A 68-year-old woman presented to her local ophthalmologist with sudden vision loss in her left eye. Subsequently, diagnosed with PCV complicated by subretinal and vitreous hemorrhages, she was referred for vitrectomy. The patient underwent a 25-gauge phacovitrectomy with internal limiting membrane peeling, sulfur hexafluoride gas tamponade, and IOL (crosslinked copolymer of 2-phenoxyethyl acrylate and ethyl acrylate) optic capture due to posterior capsular rupture. Postoperatively, best-corrected visual acuity (BCVA) improved from preoperative hand motion to counting fingers at one month, but subsequently declined due to recurrent vitreous hemorrhage despite initial improvement and subsequent anti-vascular endothelial growth factor (anti-VEGF) therapy refusal. In our region, there is no established visual acuity threshold for initiating anti-VEGF therapy in patients with neovascular age-related macular degeneration (nAMD).

**Figure 1:**
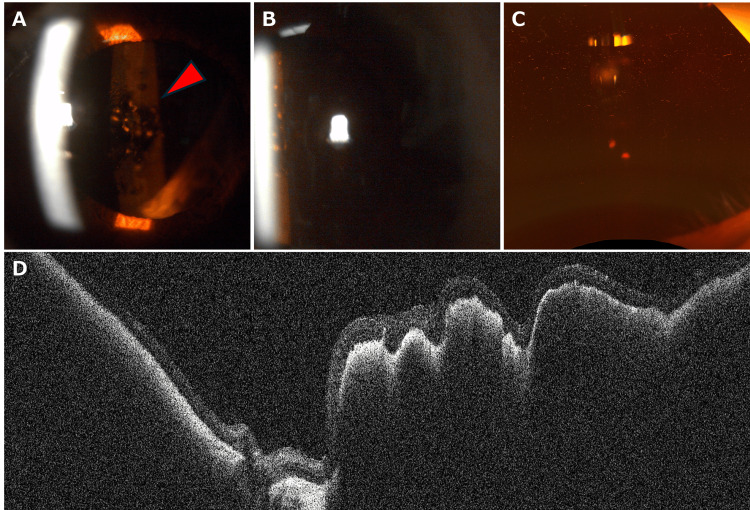
Slit-lamp examination of the anterior segment, ultra-widefield color fundus photograph, and posterior-segment optical coherence tomography of the left eye at the initial visit. At the initial visit, reddish discoloration of the iris and intraocular lens surface (red arrowhead) was observed in the left eye (A). Retroillumination reflex from the fundus was absent (B), and the fundus was not visible with ultra-widefield scanning laser ophthalmoscopy (C). However, optical coherence tomography enabled visualization of retinal structures at the initial visit (D).

Upon referral to our institution approximately three months post-operatively, the patient’s left eye BCVA was light perception, with a reddish appearance of the IOL and iris, with slit-lamp examination of the anterior segment (Figure 1A, 1B).

**Figure 2:**
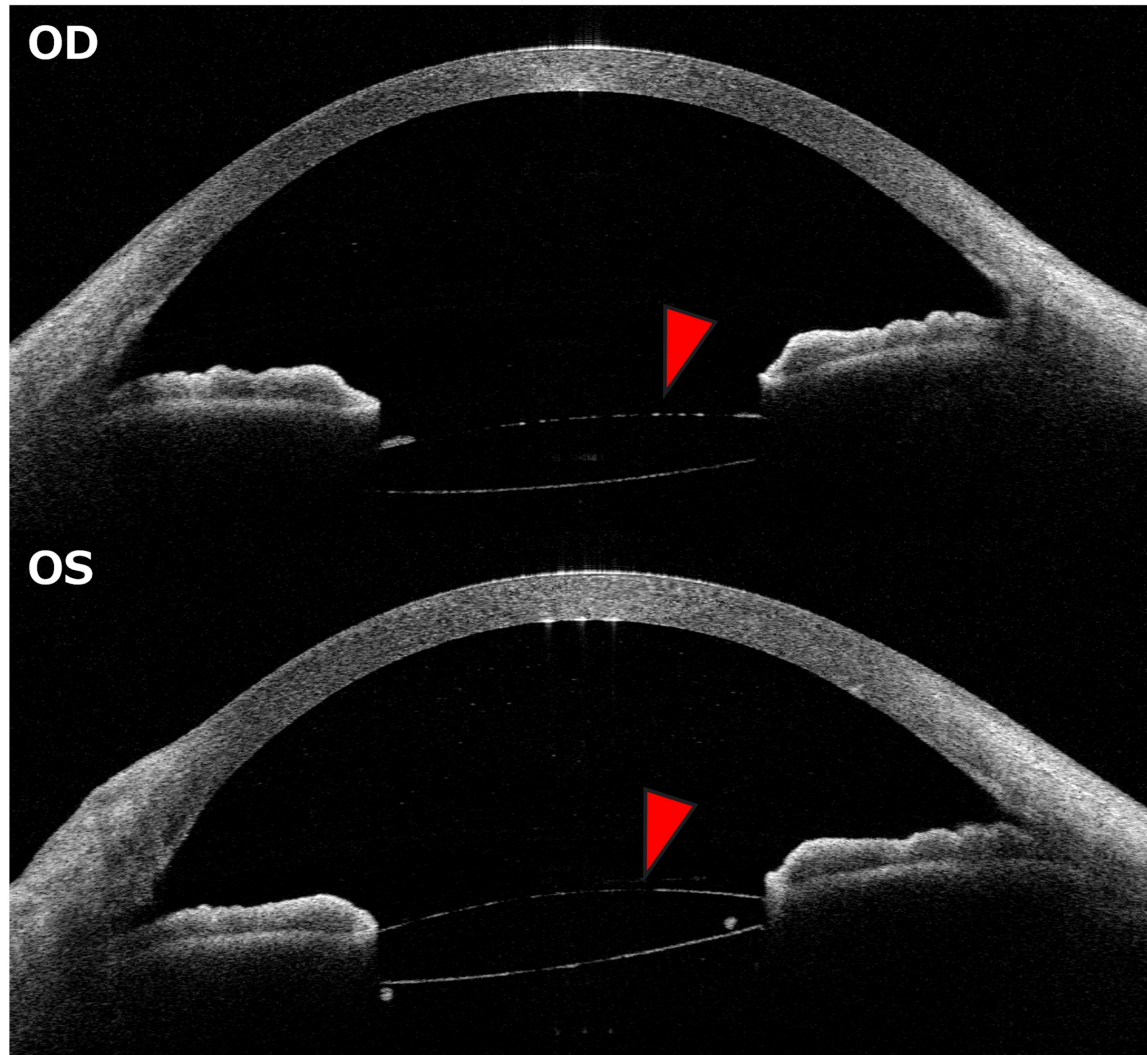
Anterior segment optical coherence tomography images at the initial visit. No opacity was observed in the optic portion of the intraocular lens in either eye (red arrowhead). Findings were comparable between the right and left eyes. OD, oculus dexter; OS, oculus sinister.

Anterior segment-optical coherence tomography (OCT) (CASIA2, Tomey Corporation, Japan) did not confirm IOL opacity and no significant differences were observed between eyes (Figure 2).

**Figure 3:**
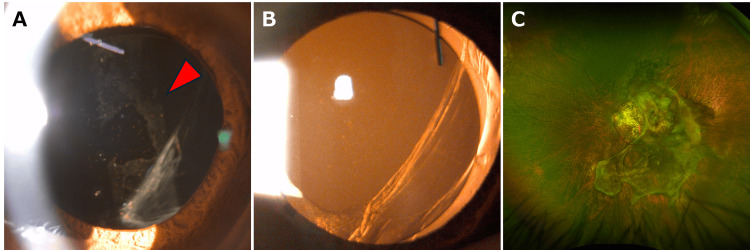
Slit-lamp examination of the anterior segment and ultra-widefield color fundus photograph of the left eye at the six-month follow-up. At the six-month follow-up, the red blood cell-coated intraocular lens had resolved (red arrowhead) (A), and retroillumination reflex from the fundus was observed (B). The fundus was visible with ultra-widefield scanning laser ophthalmoscopy (C).

Intraocular pressure was 26 mmHg in the right eye and 10 mmHg in the left eye. B-mode ultrasonography revealed a moderately hyporeflective area beneath the retina at the posterior pole, with no apparent abnormalities in the vitreous cavity. Although the left eye's posterior segment was not visible on slit-lamp examination (Figures 1A, 1C), retinal OCT (OCT-S1, Canon, Japan) revealed a significant subretinal hemorrhage (Figure 1D). A diagnosis of RBC-coated IOL secondary to PCV and massive hemorrhage was made. Treatment with intravitreal aflibercept injections at eight-week intervals led to hemorrhage resolution and gradual vision improvement to 0.04 (Snellen, 6/150) over six months. This improvement coincided with the clearance of RBC from the IOL surface (Figures 3A, 3B) and restored fundus visibility (Figure 3C).

## Discussion

This report presents detailed multimodal imaging findings of a case of RBC-coated IOL complicated by vitreous hemorrhage. When the IOL appears unclear secondary to vitreous hemorrhage, it is crucial to rule out IOL opacification. RBC-coated IOLs can be characterized by obstructed fundus visualization on slit-lamp examination, while anterior segment OCT reveals a clear IOL optic, and posterior segment OCT allows for fundus observation.

Eyes with PCV often exhibit a hemorrhagic pattern [1,3,9], and are occasionally complicated by vitreous hemorrhage [3,10]. Kim et al. reported RBC-coated IOL as a complication in 11 of 30 eyes (36.7%) undergoing vitrectomy for vitreous hemorrhage secondary to PCV [4]. These cases often involve larger subretinal hemorrhages [4], as seen in our patient. Previous reports suggest an RBC absorption period of six to 12 months on the IOL surface [4]. Our case aligns with this timeframe, with absorption occurring within six months [4]. Based on our findings and previous studies [4], observation without IOL removal may be a suitable initial management strategy for RBC-coated IOLs due to their consistent spontaneous resolution.

IOL opacification is a crucial differential diagnosis for RBC-coated IOLs. Kim et al. reported a case where an IOL was removed following a misdiagnosis of IOL opacification in a patient who was later determined to have an RBC-coated IOL via electron and light microscopy [4]. IOL opacification is typically managed by IOL removal or replacement [7]. Misdiagnosis can lead to unnecessary IOL removal [4]. IOL opacification is typically caused by calcium and/or phosphate deposits and is associated with factors like keratoplasty, diabetes, vitrectomy, gas tamponade, glaucoma, hypertension, and hydrophilic acrylic IOLs [8,11,12]. Anterior segment OCT can evaluate IOL opacification severity [13], with severe cases manifesting as a hyperreflective band [14]. In this case, the IOL was composed of a crosslinked copolymer of 2-phenoxyethyl acrylate and ethyl acrylate, a material previously reported to be resistant to IOL opacification [15]. While IOL opacification did not occur, RBC-coated IOL developed, necessitating differentiation between these two conditions. Although the causality remains unclear, posterior capsular rupture may facilitate RBC migration to the anterior chamber, potentially contributing to RBC-coated IOL formation. Therefore, preservation of the posterior capsule integrity might be crucial in preventing this complication. Currently, studies examining the OCT characteristics of IOL opacification are limited. Further investigation in this area could potentially facilitate more accurate and efficient diagnosis. Moreover, additional research is warranted to elucidate the interactions between IOL materials and fibrin or blood deposits.

In our case of RBC-coated IOL, the lens surface completely obstructed fundus visualization, but anterior segment OCT revealed a clear IOL optic, while posterior segment OCT allowed fundus observation. The different wavelengths used in these imaging modalities (1310 nm for anterior, 1060 nm for posterior) and the blood’s high absorption rate in the visible light range likely explain the discrepancy in imaging findings [16].

## Conclusions

This report documents a case of spontaneous resolution of red blood cell (RBC)-coated intraocular lenses (IOLs) following phacovitrectomy and gas tamponade in a patient with polypoidal choroidal vasculopathy complicated by vitreous hemorrhage. Our findings suggest that RBC-coated IOLs may minimally affect anterior and posterior segment optical coherence tomography imaging. Distinguishing RBC-coated IOLs from IOL opacification is crucial and warrants further investigation into the specific characteristics of RBC-coated IOLs.
